# GABA_B_ Receptor Activation Affects Eye Growth in Chickens with Visually Induced Refractive Errors

**DOI:** 10.3390/biom13030434

**Published:** 2023-02-24

**Authors:** Hong Liu, Frank Schaeffel, Zhikuan Yang, Marita Pauline Feldkaemper

**Affiliations:** 1 Section of Neurobiology of the Eye, Ophthalmic Research Institute, University of Tuebingen, 72076 Tuebingen, Germany; 2 Aier Institute of Optometry and Vision Science, Aier Eye Hospital Group, Changsha 410000, China; 3 Myopia Research Group, Institute of Molecular and Clinical Ophthalmology Basel (IOB), 4031 Basel, Switzerland; 4 Hunan Province Optometry Engineering and Technology Research Center, Changsha 410000, China; 5 Hunan Province International Cooperation Base for Optometry Science and Technology, Changsha 410000, China

**Keywords:** GABA receptor, myopia, lens induced hyperopia, dopamine, choroidal thickness, eye growth

## Abstract

This study aims to explore the role of GABA_B_ receptors in the development of deprivation myopia (DM), lens-induced myopia (LIM) and lens-induced hyperopia (LIH). Chicks were intravitreally injected with 25 µg baclofen (GABA_B_R agonist) in one eye and saline into the fellow eye. Choroidal thickness (ChT) was measured via OCT before and 2, 4, 6, 8, 24 h after injection. ChT decreased strongly at 6 and 8 h after baclofen injection and returned back to baseline level after 24 h. Moreover, chicks were monocularly treated with translucent diffusers, −7D or +7D lenses and randomly assigned to baclofen or saline treatment. DM chicks were injected daily into both eyes, while LIM and LIH chicks were monocularly injected into the lens-wearing eyes, for 4 days. Refractive error, axial length and ChT were measured before and after treatment. Dopamine and its metabolites were analyzed via HPLC. Baclofen significantly reduced the myopic shift and eye growth in DM and LIM eyes. However, it did not change ChT compared to respective saline-injected eyes. On the other hand, baclofen inhibited the hyperopic shift and choroidal thickening in LIH eyes. All the baclofen-injected eyes showed significantly lower vitreal DOPAC content. Since GABA is an inhibitory ubiquitous neurotransmitter, interfering with its signaling affects spatial retinal processing and therefore refractive error development with both diffusers and lenses.

## 1. Introduction

Myopia is a developmental eye disorder which causes blurred vision at a distance because the image is focused in front of the retina. When it progresses, it may also cause visual impairment due to chorio-retinal degeneration, glaucoma, cataract or retinal detachment. Since its prevalence continues to increase among children and adolescents in recent decades [1,2], there is urgent need to understand its mechanisms and how it could be inhibited. To gain a better understanding of its development, refractive errors can be induced in animal models with different approaches: deprivation myopia (DM), lens-induced myopia (LIM) and lens-induced hyperopia (LIH). DM is induced by reducing the spatial frequency content and contrast of the retinal image by covering the eye with a diffuser. Compensatory eye growth and myopia (LIM) can be induced when negative lenses are placed in front of the eye to impose hyperopic defocus. Conversely, eye growth inhibition and subsequent hyperopia can be induced via positive lenses [3] which impose myopic defocus. Animal models have also established the existence of local retinal control of eye growth [4]. Visual cues appear to influence axial elongation through a retina-to-retinal pigment epithelium (RPE)-to choroid-to sclera signaling cascade, leading to altered eye growth and changes in the refractive state (see [5] for a review). Choroidal thickening in response to myopic defocus and choroidal thinning in response to hyperopic defocus and deprivation was not only found in animal models but also in humans, although thickness changes in humans were much smaller than in young chicken eyes for example [6,7].

Although animal models have been used in myopia research for several decades, the underlying mechanisms are still not fully understood. Both myopia models, DM and LIM respond differentially to some interventions. High ambient lighting, for example, showed a robust protective effect against DM in infant monkeys [8], but it did not alter the compensation for LIM, nor the recovery from LIM [9]. However, since the amount of contrast loss at different spatial frequencies was not matched with diffusers and negative lenses, different results do not necessarily indicate that different mechanisms are involved. It is also striking that treatment which interferes with the development of LIM does not necessarily affect LIH in the opposite way. Dim light, for instance, attenuates both LIM and LIH in rhesus monkeys [10]. 

Gamma-Aminobutyric acid (GABA) is a widely distributed inhibitory neurotransmitter in the nervous system and plays a crucial role in brain and retina development [11] but also in retinal spatial processing [12]. GABA receptors are classified into three subtypes: GABA_A_, GABA_B_ and GABA_C_, also known as GABA_A0r_. GABA receptors localize to a large and diverse retinal cell population: GABA_A_ receptors were found in photoreceptors, horizontal cells, bipolar cells, amacrine cells and ganglion cells, GABA_B_ receptors in amacrine cells, photoreceptors and ganglion cells and GABA_A0r_ receptors localize to horizontal cells, cones and bipolar cells [13,14,15,16]. Whereas GABA acting at ionotropic type A receptors initiates fast inhibition through an increase in the chloride ion conductance, metabotropic type B receptors are coupled to a G-protein and regulate inwardly rectifying potassium and voltage-gated calcium channels and mediate slow synaptic inhibition [17], interacting with multiple neurotransmitters, including dopamine, melatonin, serotonin and glutamate [16].

It was recently shown that the modulation of GABAergic pathways influences eye growth and refractive development, but the potential targets and mechanisms are not clear. In the LIM model, the content of GABA, and GABA_A_R and GABA_C_R mRNA level were upregulated in the retina [18,19] and visual cortex [20] in guinea pigs. Antagonists of the three receptor subtypes inhibited DM in chickens [21,22]. GABA_B_R and GABA_C_R antagonists attenuated DM development in guinea pigs as well, in a dose-dependent manner [23,24]. In addition, the GABA receptor signaling pathway was found among the top 10 pathways underlying differential gene expression in the sclera of DM guinea pigs [25]. It was recently shown that GABAergic signaling participates in the anti-myopic effects of atropine in LIM in mice [26]. Meanwhile, GABAergic agents were shown to affect the DNA and glycosaminoglycan content of scleral fibroblasts in vitro, both directly and when co-cultured with myopic and hyperopic RPE/choroid tissues [27]. Based on preliminary results in guinea pigs, we will focus on the role of GABA_B_R in this study.

Dopaminergic amacrine cells synthesize and release dopamine and GABA simultaneously [28]. Dopaminergic cells establish GABAergic synapses onto AII amacrine cells, which transfer rod bipolar cell signals to cone bipolar cells. Therefore, the co-released GABA probably prevents signals from saturated rods from entering the cone pathway when the dark-adapted retina is exposed to bright illumination [29]. Dopamine (DA) mediates diverse functioning including visual signaling. The dopamine metabolites homovanillic acid (HVA) and 3,4-dihydroxyphenylacetic acid (DOPAC) are indicators of dopamine activity. Their levels decrease in myopic eyes, which may be an adaptive response to low temporal and spatial contrast in the visual image [30], and increase during recovery from induced myopia. A number of studies showed that the eye growth rate can be altered by regulating the activity of dopamine receptors [31,32]. In the brain, the perfusion of baclofen, a GABA_B_R agonist, reduced the release of dopamine in the substantia nigra and prefrontal cortex [33]. A strong and persistent suppression of dopamine release mediated by baclofen was also found in the nucleus accumbens, which was due to GABA_B_R activation on dopaminergic terminals [34]. Moreover, it was shown that baclofen suppressed light-evoked dopamine overflow in African clawed frog’s retinas, while both GABA_A_R and GABA_B_R antagonists stimulated dopamine release in light or darkness [35]. 

We hypothesize that there might be a GABA_B_R–dopamine pathway underlying the modulation of eye growth with altered visual stimuli. To verify our hypothesis, we measured the effect of baclofen on eye growth and retinal dopamine levels in chickens, using the three available ametropia models, DM, LIM and LIH. The effects of the GABABR agonists on choroidal thickness was also studied, since other studies have found an inverse association between axial eye length changes and choroidal thickness changes during experimentally induced refractive errors [6], suggesting a role for the choroid in emmetropization. 

## 2. Materials and Methods

### 2.1. Animals

White leghorn chicks at the age of one day were obtained from a local hatchery (Weiss, Kirchberg, Germany) and were reared in cages with water and food supplied ad libitum. The room temperature and humidity were managed in an appropriate range, and the light cycle was 11:13 light/dark. The chicks were of equivalent weight at the beginning of experiment 2 and 3 and baclofen injections did not influence the weight of the chickens.

### 2.2. Treatments

Experiment 1: Effect of the GABABR agonist baclofen on choroidal thickness.

R(+)-Baclofen hydrochloride (Sigma-Aldrich, Taufkirchen, Germany) dissolved in 0.9% saline solution was used in this study. The temporary effect of baclofen on choroidal thickness was explored in a short-term experiment. The baseline choroidal thickness of 7 chicks at postnatal 12 days of age (P12) was measured at 9:00 a.m., followed by intravitreal injection at 10:00 a.m. We anaesthetised the animals with ether inhalation before injection. The chicks were injected with 25 µg baclofen in a volume of 25 µL into one eye, and 25 µL saline into the other eye. The baclofen concentration was based on a study by Stone et al. (2003). We chose to inject 25 µg, a dose that was 2.5 times higher and 4 times lower than the amount they used. The reason was that a higher dose (100 µg) could potentially increase the risk of toxic side-effects and/or nonspecific effects, such as binding to other receptor types. The effects of 10 µg and 100 µg on ocular length growth described in Stone’s work were not significantly different from each other, but the mean size effect was greater at 100 µg, so we decided to inject a medium dose. Choroidal thickness was repeatedly measured at 2, 4, 6, 8 and 24 h after injection.

Experiment 2: Effect of baclofen on eye growth in deprived eyes and contralateral control eyes. 

Baseline measurements of refractive error, A-scan ultrasound and OCT were performed at P12, followed by a monocular occlusion with frosted goggles (experimental eye, diffuser) at P13. Diffusers were hand-made by frosting clear plastic with emery paper. Chicks which removed their diffusers (or lenses in experiment 3) were excluded from the experiments and their data are not reported. Chicks were randomly assigned to either saline group (*n* = 6) or baclofen group (*n* = 14). Chickens in the baclofen group were injected with 25 µg baclofen into both eyes, while those in the saline group were injected with 25 µL physiological saline solution. The un-occluded fellow eyes were also injected to explore the effect of baclofen injections on eyes with normal visual input. The injections were given daily between 10:00 and 10:30 a.m. in a well-established procedure [36], lasting for 4 days (P13–P16), after which all parameters were measured again (P17). The half-life of baclofen in the eye after intravitreal injection is not known, but a half-life time of 3–6 h has been measured after oral administration in humans [37]. The day after all the measurements were taken, chickens received another injection of either saline or baclofen at 10 a.m. to make it possible to study the immediate effects of the drug on dopamine metabolism. The animals were sacrificed 3 h later, followed by eyeball dissection and sample collection for HPLC measurements.

Experiment 3: Influence of baclofen on lens-induced myopia and lens-induced hyperopia.

**Figure 1:**
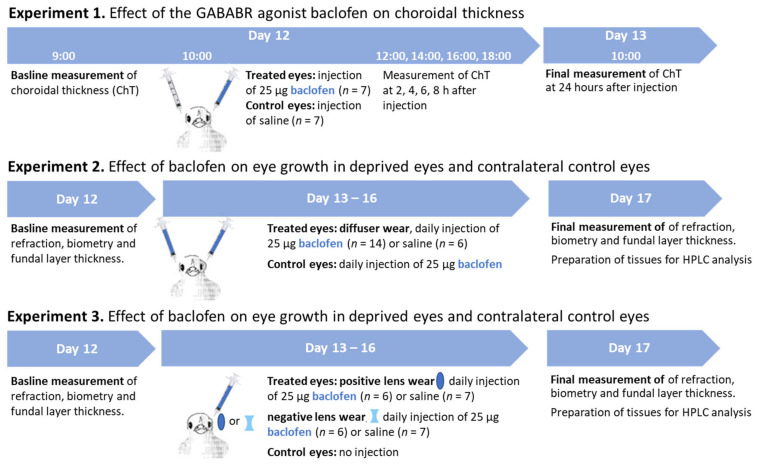
The workflow for the 3 parts of experiment.

The influence of baclofen on eye growth and dopamine metabolism in defocused eyes was detected as well. Chickens were monocularly wearing −7D (*n* = 7) or +7D (*n* = 7) lenses (Edmund optics, Mainz, Germany). Twenty-five micrograms of baclofen in 12.5 µL saline were injected into all lens-wearing eyes. Saline-injected positive (*n* = 7) or negative (*n* = 7) lens-wearing chickens were included as respective controls for the lens-treated eyes. The non-injected fellow eyes served as the internal control. The procedures of measurement, injection and sample collection were the same as in experiment 2. The workflow for the 3 parts of the experiment is displayed in Figure 1. 

### 2.3. Measurements

#### 2.3.1. Refractive Error (RE) and Ocular Biometry

RE and ocular biometrics were measured in experiment 1 before (P12) and after (P17) treatment. An automated version of infrared photoretinoscopy was used to measure the refractive error [38]. Five readings were averaged for analysis. Ocular dimensions were measured using A-scan ultrasonography with a 11 MHz probe as previously described [39]. The cornea was locally anesthetized with 2% xylocaine drops before measurements were taken. The length of the anterior chamber depth (ACD), lens thickness (LT), vitreous chamber depth (VCD) and axial length (AL) was recorded during five measurements.

#### 2.3.2. Optical Coherence Tomography (OCT)

**Figure 2:**
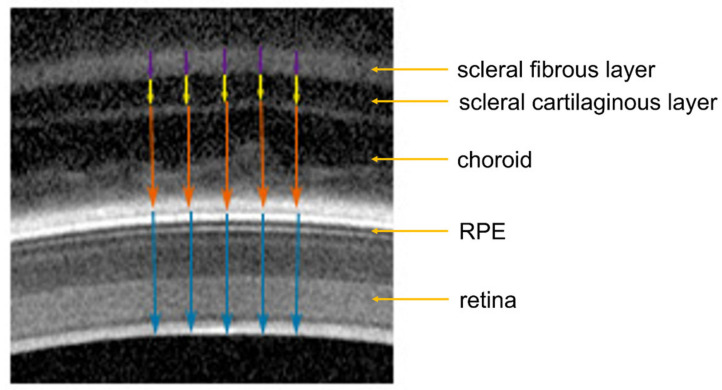
Illustration of how the chicken retinal, choroidal and scleral thickness were determined in OCT images. Blue arrows: retinal thickness; orange arrows: choroidal thickness; yellow arrows: scleral cartilaginous layer thickness; purple arrows: scleral fibrous layer thickness. RPE: retinal pigment epithelium.

Retinal thickness (RT) and choroidal thickness (ChT) were measured via OCT (Spectralis OCT, Heidelberg Engineering, Germany, resolution mode: high speed, scan angle: 30 degrees, scan type: B-scan, 768 × 496 pixels, line scan, eye tracking not engaged, scan rate of the live image 8.8 frames/sec, wavelength of measurement 1060 nm), as previously described [39]. For the measurements, alert chickens were held by hand in front of the OCT camera. To measure each time in approximately the same fundal area, the position of the chicken’s head was manually adjusted until the cornea was aligned perpendicular to the optical axis of the OCT camera and the scan of the fundal layers became visible on the screen. Optimal alignment of the chicken eye was assumed when the image of the pupil in the left screen window was centered and the scan of the fundal layers in the right window was horizontally aligned (for examples, see Figure 2). Scans became titled as soon as the eyes were not properly aligned. Repeated measurements involved re-alignment of the chicken head and eye in each case. Therefore, standard deviations reflect the repeatability of several independent alignments of the eyes. In the current study, standard deviations from repeated measurements in the same eyes ranged from 3 to 11 µm, which is considerably less than inter-individual variability (between 20 and 50 µm). Five images were analyzed for each eye. The approximate lateral distance between each of the five measurement positions within each image was 80 µm, covering a total lateral distance of about 320 µm (Figure 2).

#### 2.3.3. Sample Preparation and Measurement of Dopamine and Metabolites via HPLC

Chicks were sacrificed by an over-dose of ether and the eyes were enucleated immediately. The eyeballs were cut perpendicularly into halves with a razor blade, 1 mm posterior to the ora serrata. The anterior segment was discarded. The vitreous was removed and quickly frozen in liquid nitrogen. An 8-mm diameter circular tissue sample was cut from the central posterior eye cup using a biopsy punch as previously described [40]. The biopsy samples were consistently taken from the central area. This “central area” was defined as a circular region just above the root of the pecten. The sample was transferred to a Petri dish under the dissecting microscope with the retina facing up. Normally, the retina detached easily and could be peeled off with an ophthalmic hook. The pigment epithelium was discarded. The choroid was separated from the sclera using forceps and a hook. Any small RPE cell clusters remaining on the choroid were carefully brushed off under visual control. Since the separation efficiency of the HPLC system at the time of the analysis was not sufficiently good to analyze the choroidal catecholamine content, no such data are presented. Tissues were frozen in liquid nitrogen and stored at −80℃ for subsequent high-performance liquid chromatography (HPLC) analysis.

All vitreal samples were weighed and homogenized in 750 µL mobile phase (Thermo Fisher Scientific, Sunnyvale, CA, USA) using a tissue lyser and 5-mm stainless steel beads (TissureLyser LT, Qiagen, Hilden, Germany) at 50 Hz for 4 min. For retinal and choroidal samples, 350 µL mobile phase were added instead. Fifty microliters of retinal and choroidal homogenate were stored for protein concentration determination (BCA Protein kit for retinal samples, Micro BCA Protein kit for choroidal samples, Thermo Scientific, Rockford, IL, USA). All homogenates were centrifuged at 4 ℃ for 10 min at 14,000 *g*. The supernatant was filtered through a 0.2 µm nylon membrane filter (Thermo Scientific, Rockwood, MI, USA), and 25 µL were directly injected into the HPLC system. Samples were analyzed for catecholamine and indolamine content via HPLC (Ultimate 3000 LC with electrochemical detection ECD 3000RS, Thermo Fischer Scientific) with coulometric detection as previously described [41]. In brief, a hypersil C18 column was used (150 mm × 3 mm, 3 µm) together with a test mobile phase (Thermo Fischer Scientific) containing 10% acetonitrile and 1% phosphate buffer. The flow rate was 0.4 mL/min and the potential at the first and second electrode was set to +370 and −200 mV, respectively. Dopamine, 3,4-Dihydroxyphenylacetic acid (DOPAC), homovanillic acid (HVA, a metabolite of DOPAC), serotonin and 5′-Hydroxyindolylessigsäure (HIAA) concentrations were determined with a high reproducibility (98%). In the retina, the biogenic amine content was determined as nanogram per milligram protein (ng/mg protein), whereas in vitreous, the amount of the substances was determined relative to the wet weight (ng/100 mg wet weight).

As described previously, vitreous DOPAC levels are considered a sensitive measure of DA release from the retina, and the abundance of the DA metabolite DOPAC was therefore taken as a measure of DA activity [42,43]. The vitreal HVA level, a second DA metabolite level, was recently shown to significantly correlate with the vitreal DOPAC content [41] and might as well be suitable to document retinal dopaminergic activity.

### 2.4. Statistics

Data are shown as the mean ± SEM. A power calculation was undertaken to determine the group sizes required to achieve 80% power in observing a 1D change in refractive development (when the standard deviation (SD) is approximately 0.6D), a 0.075 mm change in axial length growth (when SD is 0.45 µm), 0.25 ng in retinal dopamine content (when SD is 0.15 ng) and ≥50 µm choroidal thickness change (when SD is 30 µm). Using a group size of 7 chicks, differences between treatment groups should become significant at the 5% level. The normal distribution of the variables was confirmed via a Shapiro–Wilk normality test. The choroidal thickness—measured at different times after injection— was compared with the respective individual thickness at baseline using a two-way ANOVA with repeated measures and Dunnett’s test for multiple comparisons. “Time” and “eye” were considered as two within-subject factors. In experiments 2 and 3, the measured ocular parameters were expressed as changes (∆, after–before). The difference between the experimental eyes and the fellow eyes were analyzed with a paired *t*-test. The difference between each two groups (exp. vs. exp., fellow vs. fellow) were compared using two-way mixed ANOVA, with “eye” and “group” being within- and between-subject factors, respectively. Sidak’s multiple comparisons test was applied in post hoc analyses. Values of *p* < 0.05 were considered as significant. The statistical analysis was performed on GraphPad prism 8 (San Diego, CA, USA). Effect size (Cohen’s d values) and 95% CI intervals were calculated using the publicly available software from pschometrica (https://www.psychometrica.de, accessed on 15 January 2023) and the formula for the comparison of samples with equal or with different sample sizes. Effect sizes (Cohen’s d) below 0.2 are considered very small to negligible, values from 0.2 to below 0.5 represent a small effect, those from 0.5 to below 0.8 represent a medium effect and values above 0.8 represent a strong effect. The calculated data for the experimental eyes and the fellow eyes were provided in the Supplementary Materials (Tables S1 and S2).

## 3. Results

### 3.1. Baclofen Temporarily Reduced Choroidal Thickness in Eyes with Normal Visual Input

**Figure 3:**
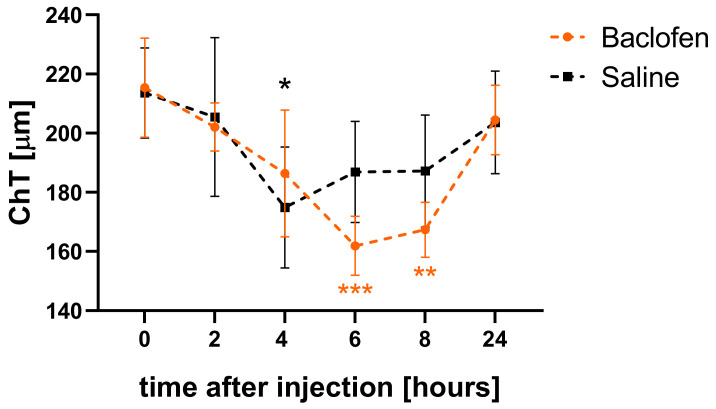
Influence of intravitreal baclofen injections on ChT changes. ChT in baclofen-injected eyes decreased significantly after 6 and 8 h compared to baseline, then recovered to baseline level after 24 h. ChT in the fellow eyes injected with saline decreased as well and reached the minimal thickness at 4 h after injection and started to recover earlier. Data are shown as mean ± SEM. Two-way repeated measures ANOVA with Dunnett’s multiple comparisons test: *: *p* < 0.05; **: *p* < 0.01; ***: *p* < 0.001.

Choroidal thickness in eyes injected with 25 µg baclofen decreased significantly after 6 and 8 h compared to the baseline level (6 h: 162.9 ± 10.0 µm vs. 215.3 ± 16.7 µm, *p* = 0.0006; 8 h: 167.4 ± 9.3 µm vs. 215.3 ± 16.7 µm, *p* = 0.002, Figure 3). After 24 h, the width of the choroid in the baclofen-injected eyes had returned to baseline (204.5 ± 11.8 µm). The contralateral eyes injected with saline also showed a decrease in choroidal thickness at 4 h (4 h vs. baseline: 174.9 ± 20.4 µm vs. 213.6 ± 15.2 µm, *p* = 0.01) but the recovery to baseline started earlier, most likely reflecting the normal diurnal cycle. At all other measurement time points, choroidal thickness was not significantly affected in these eyes. In addition to the measurement of choroidal thickness, retinal thickness (RT) was compared at baseline and 6 h after injection. In contrast to choroidal thickness, there was little change in RT and no difference between saline- and baclofen-treated eyes (delta RT: saline vs. baclofen: 5.49 ± 3.44 µm vs. 6.11 ± 2.40 µm: paired *t*-test: *p* = 0.91).

### 3.2. Baclofen Attenuates the Effects of Deprivation and Defocus on Eye Growth

**Figure 4:**
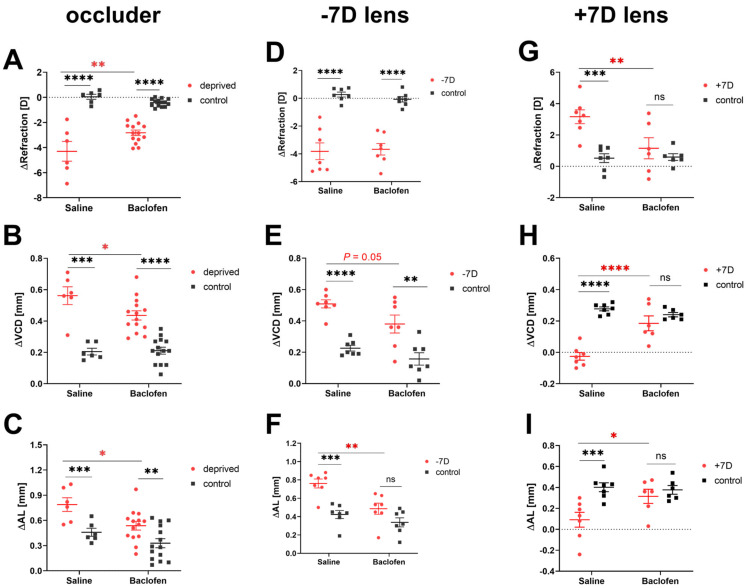
Changes in refractions and ocular biometric parameters during treatment. Control eyes of DM chicks received baclofen or saline injections, while those of LIM or LIH chicks were not injected at all. Baclofen reduced the change in refractive error in experimental eyes in DM (**A**) and LIH (**G**), but not in LIM (**D**). Baclofen retarded VCD and AL growth in DM (**B**,**C**) and LIM (**E**,**F**), and also reduced the effects of positive lenses (**H**,**I**). Data are shown as the mean ± SEM. Difference between two groups were analyzed with two-way mixed ANOVA with Sidak’s post hoc test. Binocular differences were analyzed with paired *t*-test. ns: not significant; *: *p* < 0.05; **: *p* < 0.01; ***: *p* < 0.001, ****: *p* < 0.0001.

Baclofen attenuated eye growth in all the three models used in our study, i.e., deprivation myopia, lens induced myopia and lens induced hyperopia. All diffuser-treated eyes (saline and baclofen groups) developed axial myopia, with a myopic shift in RE, a deeper VCD and longer AL compared to the un-occluded fellow eyes. However, the diffuser-treated eyes injected with baclofen became less myopic than the saline-injected eyes (saline vs. baclofen: ∆RE: −4.30 ± 0.79 D vs. −2.83 ± 0.22 D, *p* = 0.004; ∆VCD: 0.56 ± 0.06 mm vs. 0.44 ± 0.03 mm, *p* = 0.03; ∆AL: 0.79 ± 0.08 mm vs. 0.54 ± 0.05 mm, *p* = 0.02, Figure 4A–C). Likewise, all eyes wearing a −7D lens developed myopia, but those injected with baclofen had shorter eyes than those treated with saline (saline vs. baclofen: ∆VCD: 0.51 ± 0.03 mm vs. 0.38 ± 0.06 mm, *p* = 0.05; ∆AL: 0.76 ± 0.05 mm vs. 0.49 ± 0.06 mm, *p* = 0.002, Figure 3F and Figure 4E). There was no difference in ∆RE between the two groups (saline: −3.81 ± 0.60 D, baclofen: −3.67 ± 0.42 D, Figure 4D). Eyes wearing a +7D lens developed hyperopia in the saline group, with more positive RE, shorter VCDs and ALs compared to the contralateral eyes with normal vision. In addition, the effects of positive lenses were reduced in the baclofen group (+7D lens-treated eyes: saline vs. baclofen: ∆RE: +3.16 ± 0.44 D vs. +1.15 ± 0.67 D, *p* = 0.007; ∆VCD: −0.03 ± 0.03 mm vs. 0.19 ± 0.05 mm, *p* < 0.0001; ∆AL: 0.09 ± 0.07 mm vs. 0.32 ± 0.07 mm, *p* = 0.02, Figure 4G–I). There were no significant differences in the depth of the anterior chamber and lens thickness (data are shown in Supplementary Materials Figure S1).

The control eyes in the DM model injected with either saline or baclofen showed no differences in the development of refractive errors and eye lengths (Figure 4A–C). The non-injected control eyes of the lens-treated baclofen groups also developed similar refractive errors and had similar eye lengths as control eyes of the lens-treated saline groups. 

### 3.3. Effect of Baclofen on Choroidal, Retinal and Scleral Thickness

**Figure 5:**
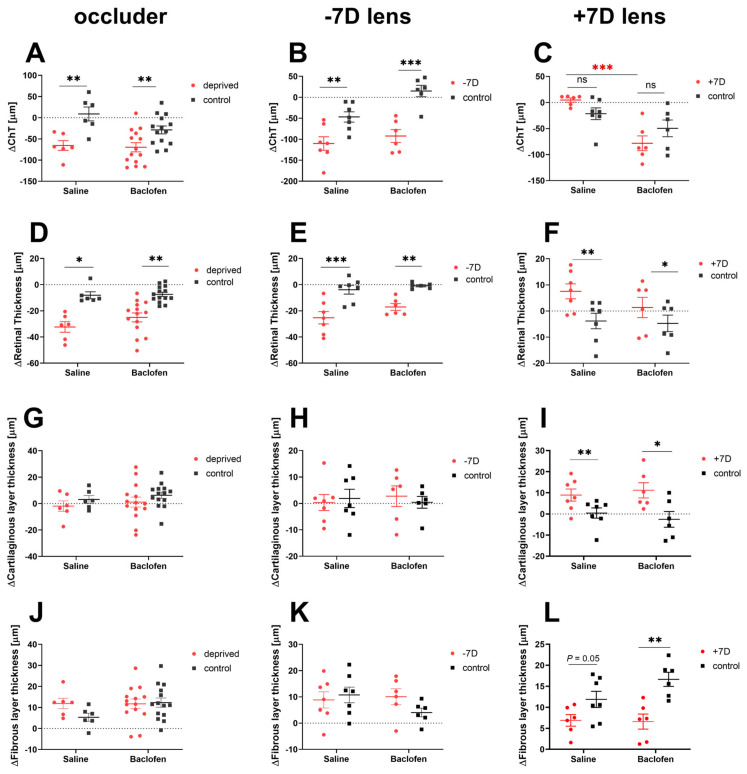
Effects of baclofen on choroidal and retinal thickness in DM, LIM and LIH. Control eyes of DM chicks received baclofen or saline injections, while those of LIM or LIH chicks were not injected at all. Choroid and retina were significantly thinner in experimental eyes than in fellow eyes in DM (**A**,**D**) and LIM (**B**,**E**); baclofen injections had no additional effect. Baclofen however thinned the choroid in LIH (**C**). Treatment with positive lenses increased retinal thickness, but there was no additional effect of baclofen vs. saline on retinal thickness (**F**). There was no difference in cartilaginous nor fibrous layer thickness between experimental and fellow eyes in DM (**G**,**J**) or LIM (**H**,**K**) groups. Positive lens-wearing eyes had a thicker cartilaginous layer (**I**) and thinner fibrous layer (**L**) than fellow eyes. Data are shown as mean ± SEM. Difference between two groups were analyzed with two-way mixed ANOVA with Sidak’s post hoc test. Binocular differences were analyzed with paired *t*-test. ns: not significant; *: *p* < 0.05; **: *p* < 0.01; ***: *p* < 0.001.

While the positive lens-treated eyes in the saline group showed a tendency to have thicker choroids than their contralateral eyes (+7D saline vs. fellow: 4.71 ± 4 µm vs. −21.53 ± 11.2 µm, *p* = 0.07), those in the baclofen group responded in the opposite way (+7D baclofen vs. fellow: −78.19 ± 14.10 µm vs. −49.60 µm ± 16.02 µm, *p* = 0.07). The effect of baclofen on choroidal thickness in +7D lens-wearing eyes was thus very different from that in eyes treated with saline and positive lenses (+7D lens: saline vs. baclofen: 4.71 ± 4 µm vs. −78.19 ± 14.10 µm, *p* = 0.0002, Figure 5C). In contrast, retinal thickness increased with positive lens treatment in both baclofen- and saline-injected +7D-lens treated eyes (+7D vs. fellow: saline: 7.54 ± 2.86 µm vs. −3.83 ± 2.96 µm, *p* = 0.003; baclofen: 1.34 ± 3.87 µm vs. −4.75 ± 3,17 µm, *p* = 0.01, Figure 5F). When myopia was induced with diffusers or negative lenses, both the choroid and retina became significantly thinner in experimental eyes than in fellow eyes (Figure 5A,B,D,E); baclofen injections had no additional effect. We also did not detect any effect of baclofen on scleral thickness. There was also no significant difference in the cartilage or fiber layer thickness between the experimental and fellow eyes in either the DM (Figure 5G,J) or LIM (Figure 5H,K) groups. The eyes that wore a positive lens had a thicker cartilage sclera than the fellow eyes (+7D vs. no lens: saline group: 8.95 ± 2.85 µm vs. 0.42 ± 2.43 µm, *p* = 0.004; baclofen group: 11.20 ± 3.62 µm vs. −2.56 ± 3.73 µm, *p* = 0.02, Figure 5I) and a significant thinner fibrous layer (+7D vs fellow: saline group: 6.89 ± 1.37 µm vs. 11.88 ± 1.92 µm, *p* = 0.05; baclofen group: 6.60 ± 1.79 µm vs 16.64 ± 1.68 µm, *p* = 0.02, Figure 5L).

### 3.4. Baclofen Reduced Retinal Dopamine Metabolism in the Three Ametropia Models

**Figure 6:**
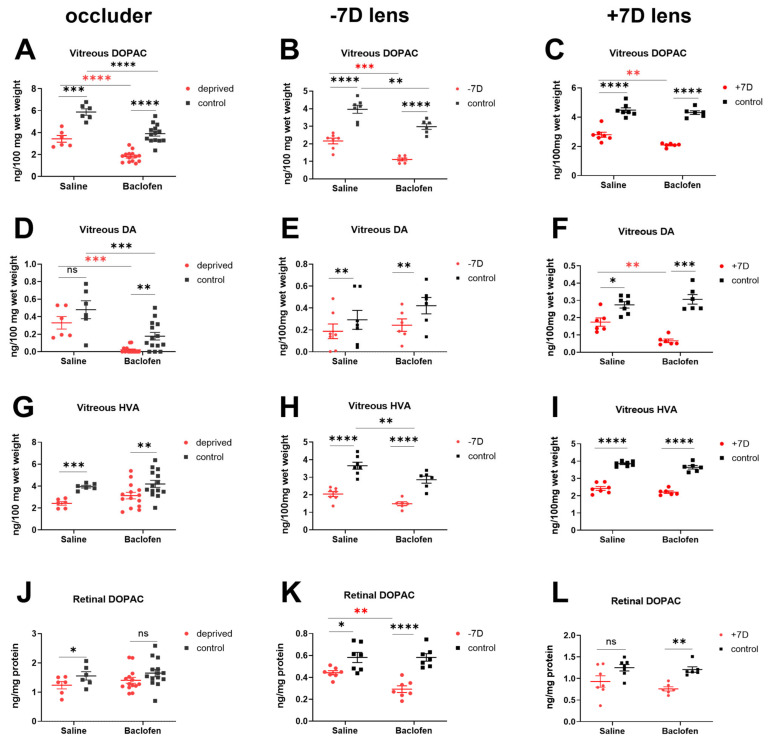
Dopamine and dopamine metabolite content. Control eyes of DM chicks received baclofen or saline injections, while those of LIM or LIH chicks were not injected at all. Baclofen significantly decreased the amount of vitreal DOPAC in all the baclofen-injected eyes (**A**–**C**), as well as vitreal dopamine level in deprived (**D**) and positive lens-treated eyes (**F**), but not in in negative lens-treated eyes (**E**). The level of vitreal HVA (**H**) and retinal DOPAC (**K**) in LIM eyes were reduced via baclofen, while there was no significant difference in deprived (**G,J**) and positive lens-treated eyes (**I,L**). Data show the mean ± SEM. Difference between two groups were analyzed with two-way mixed ANOVA with Sidak’s post hoc test. Binocular differences were analyzed with paired *t*-test. ns: not significant; *: *p* < 0.05; **: *p* < 0.01; ***: *p* < 0.001; ****: *p* < 0.0001.

Consistent with previous findings [44,45], the vitreal DOPAC level, which is considered a sensitive measure of DA release from the retina, was severely reduced in deprived and negative lens-treated eyes (Figure 6A,B). This effect was further enhanced via an injection of baclofen (saline vs. baclofen: DM: 3.43 ± 0.31 ng/100 mg vs. 1.81 ± 0.13 ng/100 mg, *p* < 0.0001; LIM: 2.16 ± 0.17 ng/100 mg vs. 1.11 ± 0.08 ng/100 mg, *p* = 0.0006, Figure 6A,B). Baclofen also reduced vitreal DOPAC levels in the fellow eyes (DM: saline-injected vs. baclofen-injected contralateral control eye: 5.87 ± 0.29 ng/100 mg vs. 3.90 ± 0.22 ng/100 mg, *p* < 0.0001; LIM: non-injected contralateral control eye saline group vs. baclofen group: 3.96 ± 0.23 ng/100 mg vs. 2.97 ± 0.16 ng/100 mg, *p* = 0.001, Figure 6A,B). In addition, vitreal dopamine levels were also lower in baclofen-injected eyes compared to saline-injected eyes (diffuser-treated eyes: saline vs. baclofen: 0.33 ± 0.07 ng/100 mg vs. 0.02 ± 0.01 ng/100 mg, *p* = 0.0004; control eyes: 0.48 ± 0.10 ng/100 mg vs. 0.18 ± 0.04 ng/100 mg, *p* = 0.0005, Figure 6D). In negative lens-treated chickens, the vitreal HVA concentration in the fellow eyes was significantly lower in the baclofen group (saline vs. baclofen: 3.66 ± 0.20 ng/100 mg vs. 2.86 ± 0.20 ng/100 mg, *p* = 0.006, Figure 6H). Additionally, the retinal DOPAC concentration in −7D lens-wearing eyes was reduced after injection of the GABABR agonist baclofen (saline vs. baclofen: 0.44 ± 0.02 ng/mg protein vs. 0.29 ± 0.03 ng/mg protein, *p* = 0.009, Figure 6K). It is important to note that dopamine metabolism was decreased in all fellow eyes in the baclofen groups, regardless of whether the fellow eyes themselves were injected with baclofen (Exp 2., fellow eyes of deprived chickens, Figure 6A,D) or were not injected (Exp. 3, fellow eyes of LIM chickens, Figure 6B,H). The contralateral effects of baclofen in the fellow eyes of LIM chicks could have possibly resulted from the systemic redistribution of the drug.

Similar to the two myopia models, retinal dopamine release and metabolism were reduced also in +7D lens-wearing eyes (Figure 6C,F,I,L), despite the fact that eye growth changed in the opposite direction. Again, administration of the GABABR agonist had an additional inhibitory effect on dopamine release and metabolism in the experimental eyes. It decreased the content of DOPAC and dopamine in the vitreous to an even larger extent (+7D lens-treated eyes: saline vs. baclofen: vitreal DOPAC: 2.80 ± 0.17 ng/100 mg vs. 2.09 ± 0.05 ng/100 mg, *p* = 0.004; vitreal dopamine: 0.17 ± 0.02 ng/100 mg vs. 0.07 ± 0.01 ng/100 mg, *p* = 0.004, Figure 6C,F). Dopamine metabolism in fellow eyes did not change. None of the treatments had a significant effect on retinal DA levels (results not shown).

The content of the indolamines serotonin and HIAA were also determined but did not change significantly and the data are therefore not shown. 

## 4. Discussion

### 4.1. Effects of the GABABR Agonist Baclofen on Axial Length, Vitreous Chamber Depth and Refractive Development

Recent studies already indicated that the GABAergic pathway might be important in the mechanisms controlling eye growth and those underlying DM [46] development, but the potential targets and mechanisms are not clear. It was suggested that “retinal GABA might act as a GO signal for myopic eye growth by interacting with other neurotransmitters, and GABA antagonists might present a novel treatment for myopia” [47]. A GABABR antagonist, CPG46381, was shown to inhibit myopia progression in deprived eyes in a dose-dependent manner in guinea pigs [24] and chickens [21] and the content of GABA was increased in LIM in guinea pigs [19]. We therefore tested whether a GABABR agonist would in return inhibit hyperopia progression. This was indeed shown in our study. Stone and coworkers also found a reduction in the myopic refractive error in deprived eyes after baclofen treatment (10 µg and 100 µg) which was not as pronounced as the effect of the antagonist CPG46381, and which was not associated with a significant change in eye length growth. We discovered in our study that baclofen (25 µg) reduced myopia progression and also the excessive ocular length growth associated with DM and LIM. Thus, the GABABR agonist attenuated the gain of both the regulatory circuits that control excessive eye length growth and those that stop eye length growth. However, baclofen was much more effective in the LIH model with respect to refractive development and eye length growth; here, the development of hyperopia was almost completely inhibited, so that the contralateral control eyes and the baclofen-injected, positive lens-wearing eyes were not different in any of the measured parameters at the end of the treatment. This result fits and complements a study by Schmid et al. [46], who showed that baclofen reduced the protective effect of normal vision against DM, attenuating the putatively altered eye growth in this experimental paradigm as well. Consistent with the study by Stone and colleagues, baclofen did not affect RE or eye length growth in non-goggled contralateral control eyes.

### 4.2. Effect of the GABABR Agonist Baclofen on Dopamine Signaling

Our results strongly support the evidence of an interaction between the GABAergic and dopaminergic system in the retina. Retinal dopamine turnover (taking vitreous DOPAC levels as measure of DA release from the retina) was found to be strongly suppressed by baclofen, regardless of if the visual input had been altered or not. We confirmed that dopamine metabolism decreases in experimental myopia [31,48] and also during hyperopia development [49]. Interestingly, baclofen injections decreased the dopamine metabolism in addition to the response to deprivation and hyperopic defocus even more. The additional effect of baclofen on DA release was also significant in the positive lens-treated eyes, but its magnitude was not as big as in DM and LIM, as we found the effect sizes for differences in DM and LIM eyes were 2.49 (95% CI: 3.72, −1.26) and 1.92 (95% CI: 3.23, 0.60), respectively, while in LIH eyes it was 0.44 (95% CI: 1.58, −0.66). Since dopamine and GABA are synthesized and released simultaneously by a group of dopaminergic amacrine cells [28], there might be an imbalanced release of dopamine and GABA, especially in myopic eyes. Dopamine and GABA are not only tightly linked in release, but also interact in function, for instance during the recovery from DM [46] and in the retinal circadian rhythm [50], the disruption of which can interfere with the emmetropization process [51]. Baclofen injections reduced dopamine and DOPAC. Interestingly, this was associated with less eye growth in the two myopia models, but more eye growth with positive lenses. Basically, baclofen seemed to reduce the gain of emmetropization. It must therefore be postulated that the GABABR agonist baclofen did not/did not solely affect ocular growth through dopaminergic signaling transduction, but interferes with other signaling pathway(s) involved in the visual control of choroidal thickness and scleral growth.

Normal expression of GABA and its key synthetic enzyme glutamate decarboxylase in the retina GAD65 is dependent on visual input, and both are decreased via dark rearing in mice [52]. Dopamine controls retinal spatial tuning by modulating receptive field sizes at multiple cellular levels [31,53]. In the myopia and hyperopia models used in our study, the retinal images were low-pass filtered via diffusers, negative or positive lenses, which had an impact on the receptive field sizes as well. Ohngemach and coworkers [42] proposed that the retinal dopamine release correlates with the amount of contrast in the retinal image. The vitreal DOPAC and dopamine content also dropped in open fellow eyes injected with baclofen (fellow eyes in the DM group), but this change was not associated with any change in refractive development and eye growth. Thus, baclofen adds to a larger number of drugs tested so far that did show an effect on refractive error development in eyes with normal visual experience. The results are in line with the other literature suggesting that treatments that lead to changes in dopamine metabolism do not affect the direction of changes in eye growth per se [42]. Low-pass filtered and defocused images, however, might trigger a signaling cascade in which the GABABR pathway is involved. The activation of GABABR attenuated the signal derived from deprivation or defocus, and thus reduced the responsive refractive development.

### 4.3. Effect of the GABABR Agonist Baclofen on Retinal and Choroidal THICKNESS

GABAB receptors have been detected in the retina [16] and RPE [54], which suggests that those tissues would be the potential targets for baclofen action. As far as we know, our study is the first report on the influence of an GABABR agonist on retinal and choroidal thickness. In general, it is already known that retinal thickness decreases during the development of myopia. A meta-analysis to evaluate the alterations in retinal OCT measurements in myopic and hyperopic patients showed that, in comparison to the controls, highly myopic eyes had a significantly thinner mean macular thickness and also retinal nerve fiber layer (RNFL), whereas hyperopic eyes had significantly thicker average peripheral RNFL than the controls [55]. We here demonstrated to our knowledge for the first time that retinal thickness in chickens treated with positive lenses increased by about 10 µm. It also significantly decreased in chicks treated with diffusers or negative lenses, an effect already known. Baclofen injections had no additional effect on retinal thickness in any of the treatment groups.

In contrast to the missing effect of baclofen on retinal thickness, we found a strong effect of the GABAB receptor agonist on choroidal thickness. Injection of baclofen thinned the choroid in control eyes with normal vision significantly at 6 and 8 h post injection, respectively, as well as in the LIH eyes at the end of the experiment. Consistent with many other studies, the choroid was thinner in DM and LIM eyes, without being further decreased by baclofen. Possibly, the choroidal thickness in the deprived eyes and the negative lens-treated eyes had already reached a minimal thickness, so baclofen could not cause a further reduction in choroidal thickness. A thicker choroid is considered to be protective against myopia development, wherein the higher blood perfusion could reduce the hypoxia in the sclera and influence scleral remodeling [56]. How baclofen affected ChT in the control and positive lens-treated eyes remains unclear. To our knowledge, it is not known so far if GABABR are expressed in the choroid. It was previously found that the GABAergic system is altered in the choroid of myopic animals, in addition to the changes in other tissues. The GABA transporter 1 (GAT-1) protein was shown to be weakly expressed in the choroid of naïve control eyes and in atropine-treated eyes, but highly expressed in the choroid of myopic mice (LIM) [27]. We can therefore only speculate if the effect is a direct receptor-mediated effect in this tissue, or an indirect effect. An indirect effect of baclofen mediated solely by changes in dopamine metabolism can be excluded, since it was shown that retinal dopamine and DOPAC levels are positively correlated with ChT in the open eyes of chickens [57] but not in ametropic eyes, as indicated by lower retinal dopamine metabolism and higher ChT in LIH eyes. 

Scleral thickness was not significantly influenced by the baclofen treatment but there is at least indirect evidence from the literature that the choroidal signals in response to baclofen are transduced to influence scleral thickness: co-culture experiments demonstrated that GABA agents, including baclofen, act on the sclera in chickens and showed that the stimulatory and inhibitory effect of GABA agents “on scleral glycosaminoglycan (GAG) and the DNA content are far greater in the presence of RPE and the choroid of myopic eyes, suggesting that the GABA agents modify either the signal from the retina to the RPE or the RPE/choroid itself or that the RPE/choroid may contain signaling molecules that modify the scleral effect of the GABA agents. The effect of the GABA agents on scleral DNA and GAG content in co-culture with posterior eye cup tissue from LIH eyes was smaller than for tissues from LIM eyes, but in the same direction.” [27].

### 4.4. Association of Reduced Efficacy of Visual Stimuli, Especially Positive Lens Wear, with Baclofen Treatment

It is striking that baclofen reduced eye growth in both directions. This suggests that the efficacy of the emmetropization process to detect and/or process optical signals is reduced. Experimental studies also suggest that the retinal GABAergic system might influence different mechanisms of visual adaptation [58]. 

Comparing the effect of atropine and baclofen on eye growth might also give us some ideas. While atropine injections increase the dopamine release, which in turn only inhibits the development of LIM and DM, but not hyperopia, baclofen injections induced a decrease in dopamine and attenuation of all growth responses. What does that mean? We speculate that GABAB receptors are involved at the very beginning of the processing of visual information, where the spatial frequency analysis is conducted, based on which all growth responses are determined—this would possibly be in the outer retina. In contrast, the atropine effect is more specific and reaches only the growth-stimulating mechanism which is inhibited. Yet another explanation could be that the retino–scleral signalling cascade is attenuated, or that the scleral target tissue becomes less responsive to such signals when GABABR are stimulated. It should be noted that there are other studies in which both LIM and LIH development were attenuated. Rearing monkey babies in low ambient light also reduced compensation for negative lenses and significantly disrupted compensation for positive lenses [59]. Furthermore, barium, a potassium channel blocker [60] and ouabain inhibition of Na/K-ATPase in the retina [61], blocked compensation to the defocus of both signs. However, it remains unclear why these different treatments affect experimentally induced ocular growth in a similar manner.

Our results might also raise the question of whether the baclofen-mediated growth responses to the visual stimuli in DM, LIM and LIH were caused by changes in local signal transmission in the retina, or whether higher centers in the brain were also affected. It was shown in several studies and a variety of species that the vision-dependent mechanisms that regulate eye growth and refractive development are located within the eye and operate in a local, regionally selective manner. It is therefore unlikely that central neural mechanisms play a primary role in refractive development, but they might be involved in the fine tuning of the response. In monkeys which were monocularly deprived for 10 days, the decreased visual input resulted in lower GABABR expression in the deprived magno- and parvo-cellular laminae of the dorsal lateral geniculate nuclei [62] and in the visual cortex [63]. It is also reported that in the rat superficial gray layer of the superior colliculus, glutamate caused the release of GABA, which then activated GABABR to depress visual responses [64]. However, more work is needed to further clarify the role of retinal GABABR activation in the visual system and visual control of eye growth.

The retina processes myopic and hyperopic defocus unequally: short periods of unrestricted vision reduce LIM quickly and dramatically, but have minimal effect on LIH [65], which means the signal for the stop of eye growth decays much slower compared with the signal for an accelerated eye growth [66,67]. A study performed on marmosets showed that interrupting defocus with unrestricted vision reduced −5D defocus compensation but enhanced +5D defocus compensation [68]. These studies show that the signaling pathways underlying the response to myopic defocus are very robust and the effects of myopic defocus are long-lasting. These altogether imply that a stimulation of GABABR might especially influence the retinal pathways activated through myopic defocus. Since baclofen attenuated the response to both negative lenses and positive lenses, one explanation could be that baclofen involves toxicity and that such toxic effects may have cumulated due to the daily injections. Toxicity could irreversibly interfere with retinal processing. However, we believe that such an explanation is less likely because the growth patterns of eyes injected with baclofen but with normal vision did not differ from those injected with saline. We therefore assume that the effect of baclofen injections in our study is due to a specific, nontoxic effect on the retinal circuits partial to myopic defocus. 

Up to now, the pharmacology of lens-induced hyperopia has received little attention. The glucagonergic antagonist des-His1-Glu9-glucagon amide has been shown to inhibit the development of hyperopia in chicks, albeit only within a narrow concentration range and by no more than 50% [69]. In addition, chicks treated with the nNOS inhibitor (neuronal isoform of nitric oxide synthase) Nω-propyl-L-arginine did not become hyperopic in response to the myopic defocus induced by positive lenses and showed a significant inhibition in the choroidal response [70]. Furthermore, chicks intravitreally injected with the D isomer of Æ-aminoadipic acid, inhibiting the retinal OFF response, showed a reduced refractive compensation to positive lenses [71]. Thus, to our knowledge, the GABA agonist baclofen is now the fourth pharmacological treatment that inhibits experimentally induced hyperopia. 

It is already known that the effects of GABA in the retina change with age in ferrets [72]. In particular, GABA plays a very different role in early retinal development, long before the onset of visual function, as compared to its function in the mature retina. We can therefore only comment on the effect of GABAergic modulation at the time interval tested in our study.

### 4.5. Limitations of the Study

There are several limitations in our study. Firstly, we only tested the influence of a GABABR agonist. Additional studies on the effect of GABABR antagonists on dopamine metabolism and choroidal thickness changes are intended, because the antagonists tested so far seem to be more potent in reducing the accelerated eye growth during myopia development and could therefore be interesting as potential drugs for myopia control. Secondly, we did not test different concentrations of baclofen. It was shown that high doses of the GABABR agonist baclofen—after intraperitoneal injection—activated GABAAR as well [73]. However, since a previous study demonstrated that eye growth in chicks injected with 10 µg or 100 µg baclofen were different from those injected with a GABAAR agonist [21], we believe that the dosage of 25 µg in our study was not too high and that the measured effects were specific for GABABR action. Lastly, in the study described here, the same amount of baclofen (25 µg) was injected in all experiments, but the volume in which the drug was dissolved was varied (25 µL in Exp. 1 and Exp. 2 versus 12.5 µL in Exp. 3). The resulting difference in baclofen concentration could have affected the pharmacokinetics, i.e., the time required for baclofen to bind to the receptor, as well as the binding specificity. Since the results of the different ametropia models (DM, LIM, LIH) were analyzed separately and were compared to the matched control groups, we nevertheless think that our conclusion was not affected by this difference in experimental design.

## 5. Conclusions

In conclusion, we found that growth changes occurring in various ametropia models were attenuated after administration of the GABABR agonist baclofen, with ocular growth slowed via diffusers or negative lenses and growth inhibition reduced via positive lenses. Baclofen might therefore interact with both the growth-inhibitory and growth-promoting pathways of the emmetropization feedback loop. In addition, treatment with baclofen was associated with short-term reduced choroidal thickness in eyes with normal vision and a long-term reduction in eyes with positive lenses. However, the postulated inverse correlation between choroidal thickness and axial length changes was not observed in myopic eyes treated with baclofen. Dopamine turnover in the retina was also greatly reduced. Because GABA is a ubiquitous transmitter, disruption of its signaling might not only reduce the development of myopia but also affect visual function. All these factors need to be carefully investigated before agents acting on GABABR can be proposed as a new approach to influence myopia development.

## Data Availability

All data reported are provided in the text or in the figures.

## Supplementary Materials

The following supporting information can be downloaded at: https://www.mdpi.com/article/10.3390/biom13030434/s1, Figure S1: Changes in anterior chamber depth (ACD) and lens thickness during the treatment; Table S1: Statistical Analysis of the effect of baclofen in the experimental eyes; Table S2: Statistical Analysis of the effect of baclofen in the open fellow eyes.
