# Supplementary material for: GABAB Receptor Activation Affects Eye Growth in Chickens with Visually Induced Refractive Errors
Source: Biomolecules. 2023 Feb 24;13(3):434. doi: 10.3390/biom13030434 (PMC10046083; doi:10.3390/biom13030434)
Supplement: Supplementary file 1 [file biomolecules-13-00434-s001.zip › biomolecules-2118009-supplementary.pdf]

Table S1. Statistical Analysis of the effect of baclofen in the experimental eyes

treatment	parameters	Predicted (LS) mean difference	95% CI of difference	Effect size (Cohen's *d*)	95% CI of effect size
Deprivation	RE (D)	-1.47	[-2.50, -0.44]	1.20	[0.18, 2.23]
	VCD (mm)	0.13	[0.01, 0.24]	1.01	[0.00, 2.01]
	AL (mm)	0.25	[0.04, 0.47]	**1.30**	**[0.26, 2.33]**
	ChT	4.15	[-37.86, 46.15]	0.11	[-0.85, 1.07]
	RT	-7.30	[-18.16, 3.57]	0.61	[-0.37, 1.58]
	CLT	-3.05	[-15.67, 9.57]	0.23	[-0.73, 1.19]
	FLT	0.16	[-8.59, 8.92]	0.02	[-0.94, 0.98]
	Vitreal DOPAC	1.61	[0.822, 2.41]	**2.78**	**[1.49, 4.06]**
	Vitreal DA	0.31	[0.14, 0.48]	**3.19**	**[1.81, 4.56]**
	Vitreal HVA	-0.71	[-1.85, 0.43]	0.73	[-0.25, 1.71]
	Retinal DOPAC	-0.17	[-0.63 to 0.29	0.46	[-0.51, 1.43]
-7 D lens wear	RE (D)	-0.15	[-1.46, 1.17]	0.10	[-0.95, 1.15]
	VCD (mm)	0.13	[0.00, 0.26]	1.11	[-0.02, 2.24]
	AL (mm)	0.28	[0.10, 0.45]	1.85	[-0.60, 3.10]
	ChT	-17.83	[-67.01, 31.34]	0.44	[-0.66, 1.54]
	RT	-8.22	-19.71, 3.2]	0.81	[-0.32, 1.94]
	CLT	-2.39	[-13.38, 8.60]	0.27	[-0.82, 1.37]
	FLT	-1.22	[-10.80, 8.36]	0.16	[-0.94, 1.25]
	Vitreal DOPAC	1.05	[0.47, 1.64]	**3.01**	**[1.42, 4.60]**
	Vitreal DA	-0.06	[-0.30, 0.19]	0.35	[-0.75, 1.45]
	Vitreal HVA	0.55	[-0.02, 1.13]	**1.67**	**[0.41, 2.94]**
	Retinal DOPAC	0.15	[0.04 to 0.26]	**2.26**	**[0.92, 3.60]**
+7D lens wear	RE (D)	2.00	[0.54 to 3.47]	**1.43**	**[0.21, 2.65]**
	VCD (mm)	-0.21	[-0.30, -0.12]	**2.39**	**[0.96, 3.81]**
	AL (mm)	-0.22	[-0.42, -0.03]	1.27	[0.08, 2.46]
	ChT	84.90	[42.95, 126.90]	**3.27**	**[1.54, 5.00]**
	RT	6.19	[-4.67, 17.08]	0.73	[-0.4, 1.86]
	CLT	-2.25	[-12.89, 8.39]	0.28	[-0.82, 1.37]
	FLT	0.29	[-5.72, 6.30]	0.07	[- 1.06, 1.21]
	Vitreal DOPAC	0.71	[0.22, 1.19]	**2.01**	**[0.67, 3.34]**

	Vitreal DA	0.11	[0.04, 0.18]	**2.38**	**[0.90, 3.86]**
	Vitreal HVA	0.22	[-0.08, 0.51]	0.92	[-0.229, 2.06]
	Retinal DOPAC	0.17	[-0.13, 0.48]	0.64	[-0.48, 1.76]

Difference (Sidak's multiple comparisons post-hoc test) and effect size (Cohen`s d) with the respective 95% coincidence intervals: saline group versus baclofen group.

Table S2. Statistical Analysis of the effect of baclofen in the open fellow eyes

treatment	parameters	Predicted (LS) mean difference	95% CI of difference	Effect size (Cohen's *d*)	95% CI of effect size
Deprivation	RE (D)	0.46	[-0.57, 1.49]	**1.29**	**[0.26, 2.33]**
	VCD (mm)	-0.01	[-0.12, 0.11]	0.08	[-0.88, 1.03]
	AL (mm)	0.13	[-0.08, 0.34]	0.71	[-0.27, 1.69]
	ChT	37.62	[-4.38, 79.62]	1.03	[0.02, 2.04]
	RT	-0.71	[-11.57, 10.16]	0.11	[-0.84, 1.07]
	CLT	-3.17	[-15.79, 9.45]	0.37	[-0.60, 1.33]
	FLT	-6.98	[-15.73, 1.77]	0.94	[-0.06, 1.94]
	Vitreal DOPAC	1.97	[1.18, 2.77]	**2.49**	**[1.26, 3.72]**
	Vitreal DA	0.30	[0.13, 0.48]	**1.58**	**[0.51, 2.65]**
	Vitreal HVA	-0.25	[-1.39, 0.89]	0.24	[-0.72, 1.20]
	Retinal DOPAC	-0.10	[-0.55, 0.36]	0.22	[-0.74, 1.18]
-7 D lens wear	RE (D)	0.34	[-0.97, 1.65]	0.70	[-0.38, 1.78]
	VCD (mm)	0.07	[-0.06, 0.20]	0.84	[-0.25, 1.93]
	AL (mm)	0.09	[-0.09, 0.26]	0.70	[-0.38, 1.78]
	ChT	-61.89	[-111.1, -12.71]	**1.89**	**[0.52, 3.20]**
	RT	-3.02	[-14.51, 8.48]	0.45	[-0.66, 1.55]
	CLT	1.47	[-9.52, 12.47]	0.19	[-0.90, 1.28]
	FLT	6.71	[-2.87, 16.29]	1.04	[-0.12, 2.21]
	Vitreal DOPAC	0.99	[0.40 to 1.58]	**1.92**	**[0.60, 3.23]**
	Vitreal DA	-0.13	[-0.38, 0.12]	0.62	[-0.50, 1.73]
	Vitreal HVA	0.80	[0.22, 1.38]	**1.86**	**[0.31, 2.79]**
	Retinal DOPAC	0.00	[-0.11, 0.11]	0.003	[-1.04, 1.05]
	RE (D)	-0.07	[-1.53, 1.39]	0.002	[-1.09, 1.09]
	VCD (mm)	0.04	[-0.06, 0.13]	1.12	[0.05, 2.29]

+7D lens wear					
	AL (mm)	0.02	[-0.17, 0.22]	0.23	[-0.86, 1.32]
	ChT	28.08	[-12.64, 68.80]	0.91	[-0.24, 0.24]
	RT	0.92	[-9.97, 11.80]	0.12	[-0.97, 1.21]
	CLT	2.99	[-7.66, 13.63]	0.38	[-0.72, 1.49]
	FLT	-4.77	[-10.56, 1.02]	1.02	[-0.14, 2.18]
	Vitreal DOPAC	0.17	[-0.32, 0.65]	0.44	[-0.66, 1.55]
	Vitreal DA	-0.03	[-0.10, 0.04]	0.55	[-0.56, 1.66]
	Vitreal HVA	0.21	[-0.09, 0.50]	1.04	[-0.12, 2.21]
	Retinal DOPAC	0.04	[-0.26, 0.35]	0.23	[-0.86, 1.33]

Difference (Sidak's multiple comparisons post-hoc test) and effect size (Cohen`s d) with the respective 95% coincidence intervals: saline group versus baclofen group.

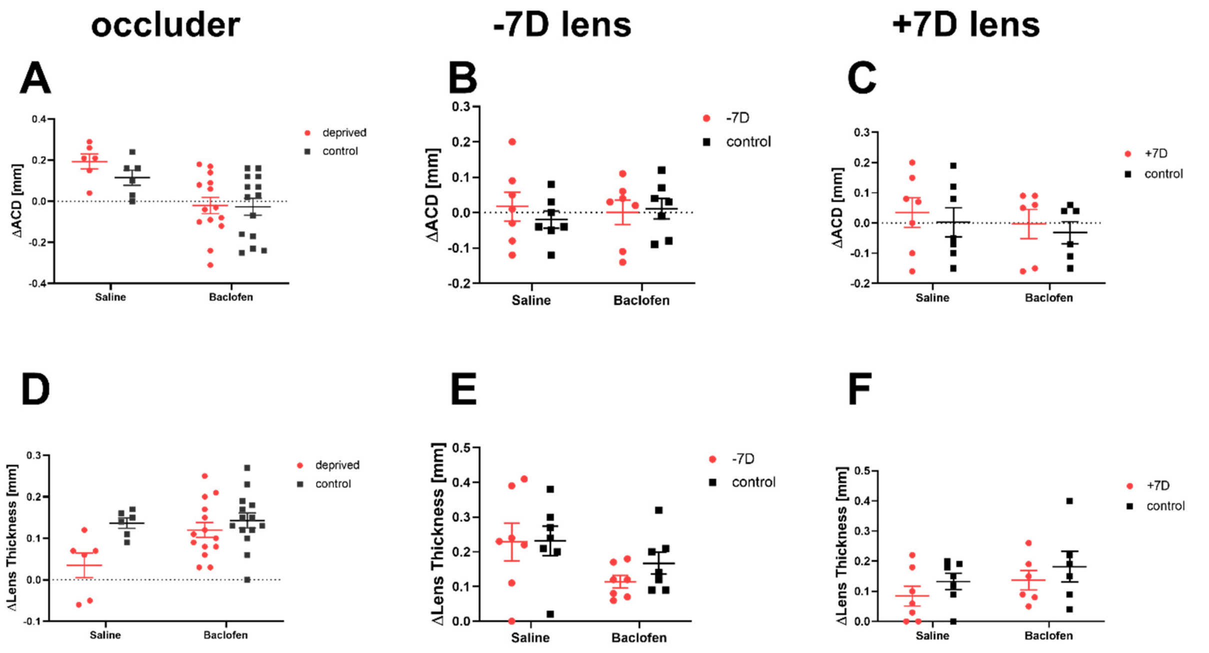

Figure S1. Changes in anterior chamber depth (ACD) and lens thickness during the treatment. Baclofen has no effect on anterior chamber and lens development in DM (A, D), LIM (B, E) or LIH (C, F). Data are shown as the mean ± SEM. Difference between two groups were analyzed with two-way mixed ANOVA with Sidak's post hoc test. Binocular differences were analyzed with paired t-tests.
